# Discrepancies between current displayed and auto‐logged glucose values in FreeStyle Libre 3: Implications for clinical interpretation

**DOI:** 10.1111/dom.70140

**Published:** 2025-09-18

**Authors:** Lilian Witthauer, Camilo Mendez, José Garcia‐Tirado, Manuel Eichenlaub, Delia Waldenmaier, Stefan Pleus, Guido Freckmann

**Affiliations:** ^1^ Department of Diabetes, Endocrinology, Nutritional Medicine and Metabolism, Inselspital, Bern University Hospital University of Bern Bern Switzerland; ^2^ Diabetes Center Berne Bern Switzerland; ^3^ Graduate School for Cellular and Biomedical Sciences University of Bern Bern Switzerland; ^4^ Institut für Diabetes‐Technologie Forschungs‐ und Entwicklungsgesellschaft mbH an der Universität Ulm Ulm Germany

**Keywords:** clinical trial, continuous glucose monitoring (CGM), glycaemic control, type 1 diabetes

## Abstract

**Aims:**

The FreeStyle Libre 3 (FSL3) continuous glucose monitoring (CGM) system provides both auto‐logged glucose values (AL) and current displayed glucose values (CUR). These values are often assumed to be interchangeable; however, discrepancies and their clinical relevance remain underexplored.

**Materials and methods:**

Data from a 15‐day study in 24 study participants wearing FSL3 were analysed, including three in‐clinic sessions with glycaemic excursions during which CUR values were retrieved every 15 min by study personnel. Paired AL and CUR readings were compared using descriptive statistics, Wilcoxon signed‐rank tests, and mean absolute relative difference (MARD) calculations. Display errors defined as instances where the app showed an error message instead of a CUR value were analysed using linear mixed‐effects models to assess associations with glucose level and rate of change (RoC).

**Results:**

CUR values were comparable to AL in general (mean difference: −1.2 ± 6.4 mg/dL), but slightly lower in the hypoglycaemic range. Discrepancies exceeding ±10 mg/dL occurred in about 10% of cases. MARD was comparable between AL (9.7%) and CUR (10.1%), with greater deviation in hypoglycaemia. Display errors (3.9%) occurred more often at higher glucose levels (mean AL difference: +91.9 mg/dL) and during rapid fluctuations (mean absolute RoC difference: +1.52 mg/dL/min; both *p* < 0.001).

**Conclusions:**

Although differences between AL and CUR were generally small, they were systematic and more pronounced in critical contexts like hypoglycaemia and rapid glucose change. Recognising these patterns may improve CGM data interpretation and alignment between user actions, provider decisions, and automated systems. These differences can reclassify readings across clinical thresholds, affecting patient–clinician alignment despite small average biases.

## 1 INTRODUCTION

Continuous glucose monitoring (CGM) systems, such as the FreeStyle Libre 3 (FSL3; Abbott Diabetes Care Inc., Alameda, California), have significantly advanced diabetes management by providing real‐time glucose data.^1^, ^2^ The FSL3 has been validated for use in clinical practice, with studies showing that it delivers accurate glucose readings.^3^, ^4^, ^5^ This system provides two primary data streams: auto‐logged glucose values (AL), which are recorded automatically at 5‐min intervals and graphically displayed in the app or a dedicated reader, and current glucose values (CUR), which are shown numerically when the user opens the smartphone application or activates the reader, and can update as frequently as once per minute. Although active scanning is no longer required as in previous device generations, CUR values fulfil the same function by providing users with an up‐to‐date glucose reading. Importantly, neither AL nor CUR values can be displayed in numerical form for past time points via the app or the reader. While AL values are typically extracted by diabetes professionals through the manufacturer's data management platform to support retrospective trend analysis and the calculation of CGM‐derived metrics, CUR values are generally consulted by individuals with diabetes during key decision‐making moments, such as insulin dosing or meals. Additionally, there are situations in which CUR values are temporarily unavailable and an error is displayed, while a complete set of AL values can be retrieved retrospectively.

Understanding the differences between AL and CUR data streams, and the conditions in which CUR values may be temporarily unavailable is crucial, as discrepancies may impact diabetes management decisions^6^ and have the potential to contribute to user confusion. Therefore, this post hoc analysis aims to quantify and characterise systematic differences between AL and CUR values provided by FSL3, and to examine the conditions under which display errors occur.

## 2 MATERIALS AND METHODS

### 2.1 Dataset

FSL3 data were obtained from the dataset collected during the study “Clinical performance evaluation of three continuous glucose monitoring systems in adults with diabetes mellitus using a newly proposed testing procedure” (DRKS00033697). The study spanned 15 calendar days, during which participants wore CGM sensors in a predominantly free‐living setting. Each participant inserted the FSL3 sensor on day 1, which remained in place until the end of its functional life on day 15. FSL3 was used in conjunction with an Android‐based smart device on which the FSL3 application was installed.

Each participant completed three in‐clinic sessions with induced glycaemic excursions, which were conducted on study days 2, 5, and 15. During these sessions, reference glucose concentrations were measured every 15 min using three methods: venous glucose levels (YSI 2300 STAT PLUS and COBAS INTEGRA 400 plus analysers) and capillary blood glucose levels (Contour Next monitoring system). In parallel, CUR values were scheduled to be retrieved every 15 min by study personnel by opening the corresponding smartphone application. With FreeStyle Libre 3, opening the smartphone app automatically displays the current glucose; no manual NFC scan is required (unlike Libre/Libre 2). Each scheduled retrieval attempt was documented using one of the following status labels:

- ‘Successful’: A value was successfully numerically displayed in the app.
- ‘Display Error’: The app displayed an error ‘Sensorfehler’ (sensor error) instead of a CUR value.

Participants were permitted to view their glucose values at their own discretion throughout the study. However, only values retrieved by study personnel during in‐clinic sessions were included in the present analysis to avoid bias from variability in individual access behaviour. AL and CUR timestamps and CGM readings were exported using the manufacturer's data management platform, which is routinely available to individuals with diabetes and clinicians. Additional details regarding the study design and in‐clinic session protocol are provided in Eichenlaub et al.^5^ and Link et al.^7^

### 2.2 Data preprocessing and analysis

All data were processed using Python (v3.11.7) with the pandas, NumPy, SciPy, scikit‐learn, matplotlib, and statsmodels libraries. Timestamps were converted to datetime format and sorted chronologically for each participant. Retrieval outcomes were extracted from the in‐clinic session documentation. For attempts labelled as ‘Successful’, missing CUR values occasionally occurred due to slight timing mismatches between the documented retrieval status and the timestamp of the exported CUR value. In such cases, the nearest available CUR value within a ±2‐minute window was used for imputation. Successful retrievals with no matching CUR value within this window were excluded from the analysis.

To evaluate agreement between CUR and AL, all time points where both values were available within a 5‐minute window and a ‘Successful’ status were selected. The pointwise difference between CUR and AL glucose values was analysed across glycaemic ranges defined by AL levels: hypoglycaemic (<70 mg/dL), euglycaemic (70–180 mg/dL), and hyperglycaemic (>180 mg/dL), as well as overall. Descriptive statistics, including mean and standard deviation, were computed. The Shapiro–Wilk test was used to assess the normality of difference distributions, and the Wilcoxon signed‐rank test was applied to evaluate paired differences.

To assess point accuracy of AL and CUR values with respect to the reference measurements, the mean absolute relative difference (MARD) was calculated between both AL and CUR values and blood glucose reference measurements across the defined glycaemic ranges. Capillary glucose served as the primary comparator, consistent with recent IFCC WG‐CGM recommendations^8^; venous YSI analyses are provided in Appendix S1.

To identify conditions associated with display errors, AL values and their absolute rate of change (RoC) were extracted at the time of each retrieval attempt. RoC was calculated as the absolute change in AL values divided by the elapsed time in minutes between consecutive AL readings. Differences in glucose levels and RoC between attempts labelled ‘Successful’ and ‘Display Error’ were assessed using linear mixed‐effects models, with participant ID included as a random effect to account for repeated measures.

To analyse peak glucose differences between data streams, the maximum and minimum values from AL and CUR were identified for each participant and session. Only pairs occurring within ±5 min of each other were included to ensure valid temporal alignment.

## 3 RESULTS

### 3.1 Overview of CGM data

The dataset analysed in this study includes data from 24 participants. A total of 2088 CUR retrieval attempts were performed by study personnel during the in‐clinic sessions. Of these, 1950 attempts (93%) were successful (‘Successful’ status within ±2 min), resulting in valid current displayed values. Seventy‐nine attempts (4%) resulted in display errors (‘Display Error’), and 59 (3%) attempts were marked as not performed typically due to prior sensor removal.

**FIGURE 1:**
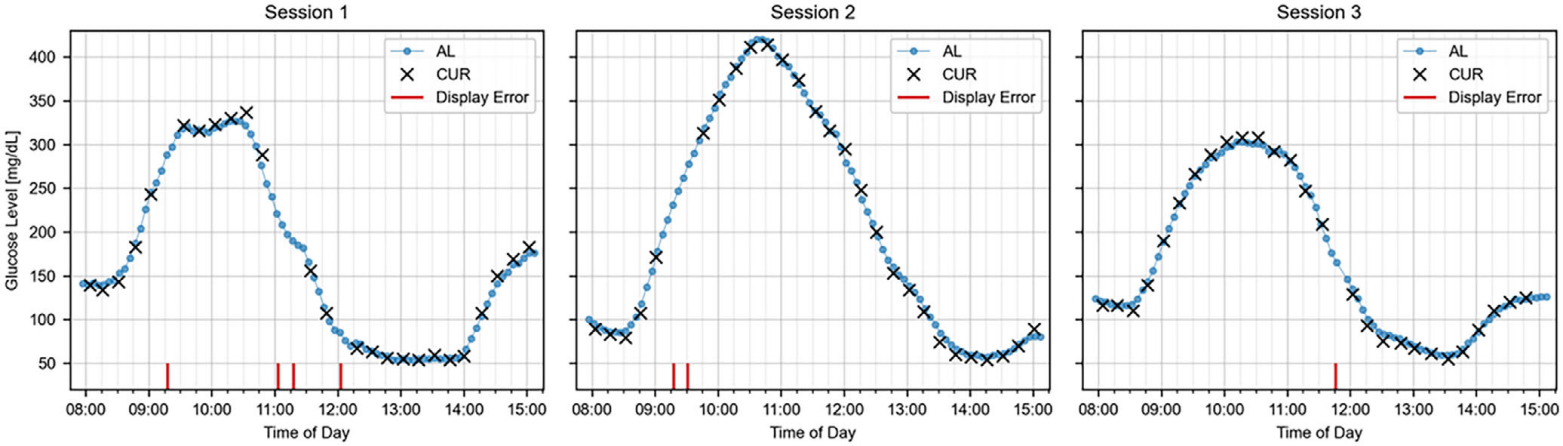
CGM data from an example participant during in‐clinic sessions. Comparison of AL glucose values (blue circles) and CUR glucose values (black crosses) and display errors (red).

Figure 1 presents the CGM data from an example participant, illustrating the comparison between AL and CUR values from in‐clinic sessions and highlighting instances of display errors. These events, which occur when the app is unable to display a CUR value, underscore the challenges of obtaining accurate data during dynamic glucose fluctuations.

### 3.2 Current displayed versus auto‐logged values

**FIGURE 2:**
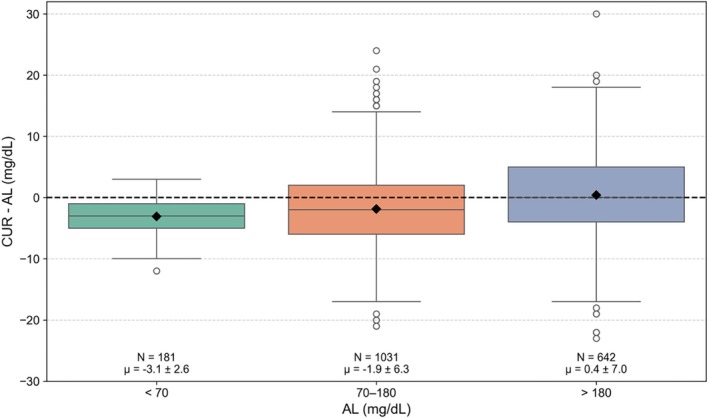
Distribution of differences between CUR and AL glucose values across glycaemic ranges: Hypoglycaemic (<70 mg/dL), euglycaemic (70–180 mg/dL), and hyperglycaemic (>180 mg/dL), as well as overall. Boxes represent the interquartile range (25th to 75th percentile) with medians as horizontal lines. Whiskers extend to 1.5 times the interquartile range; outliers are shown as individual circles. Diamonds denote mean values. The dashed line indicates zero bias. Mean, standard deviation, and number of points are annotated for each category.

The distribution of differences between paired CUR and AL glucose values is shown in Figure 2, stratified by glycaemic range (from AL; pairs matched within ±5 min; mean |Δ*t*| = 1.22 ± 0.76 min). Across hypoglycaemia (<70 mg/dL) and euglycaemia (70–180 mg/dL), CUR values were slightly lower than AL; in hyperglycaemia (>180 mg/dL), CUR values tended to be slightly higher. The mean (±SD) differences were −3.1 ± 2.6 mg/dL in the hypoglycaemic range (<70 mg/dL), −1.9 ± 6.3 mg/dL in the euglycaemic range (70–180 mg/dL), and 0.4 ± 7.0 mg/dL in the hyperglycaemic range (>180 mg/dL). Overall, the mean difference was −1.2 ± 6.4 mg/dL, indicating a small but consistent negative bias of CUR values relative to AL ones. While the variability increased with glucose concentration, the average differences remained clinically minor.

To estimate the frequency of large discrepancies between CUR and AL values, the 5th and 95th percentiles of their paired differences were calculated. Assuming 10 user‐initiated value checks per day, this corresponds to the range within which 90% of AL‐CUR differences are expected, indicating that approximately one current displayed value per day may fall outside this interval. In the hypoglycaemic range (<70 mg/dL), 90% of differences fell between −8 and +1 mg/dL; in the euglycaemic range (70–180 mg/dL), between −11 and +9 mg/dL; and in the hyperglycaemic range (>180 mg/dL), between −10 and +12 mg/dL. Overall, 90% of paired differences were within −11 to +10 mg/dL. The relationship between the difference between CUR and AL and the RoC is illustrated in Appendix S1.

To assess reclassification at clinical decision thresholds, we defined threshold discordance as paired readings falling on opposite sides of 70 or 180 mg/dL. Discordance was uncommon but present: CUR < 70 mg/dL with AL ≥ 70 mg/dL in 2.9% of all pairs and AL < 70 mg/dL with CUR ≥ 70 in 0.1% of all pairs; CUR > 180 mg/dL with AL ≤ 180 mg/dL in 0.5% of all pairs and AL > 180 mg/dL with CUR ≤ 180 mg/dL in 0.9% of all pairs. Consistent with this, out of the paired CUR and AL values, fewer CUR than AL values were within the 70–180 mg/dL range (53.2% vs. 55.6%; Δ −2.4 percentage points).

The Shapiro–Wilk test indicated that the distribution of differences between CUR and AL values was not normally distributed across all glycaemic ranges and overall (*p* < 0.01 in all cases). The Wilcoxon signed‐rank test indicated significant differences between CUR and AL values in the hypoglycaemic and euglycaemic ranges (*p* < 0.0001), but not in the hyperglycaemic range (*p* = 0.195). These results suggest a systematic negative bias in CUR values at lower glucose levels, while differences in hyperglycaemia appear more variable and less directionally consistent. The overall difference was also statistically significant, largely reflecting shifts observed in the lower glycaemic ranges.

**FIGURE 3:**
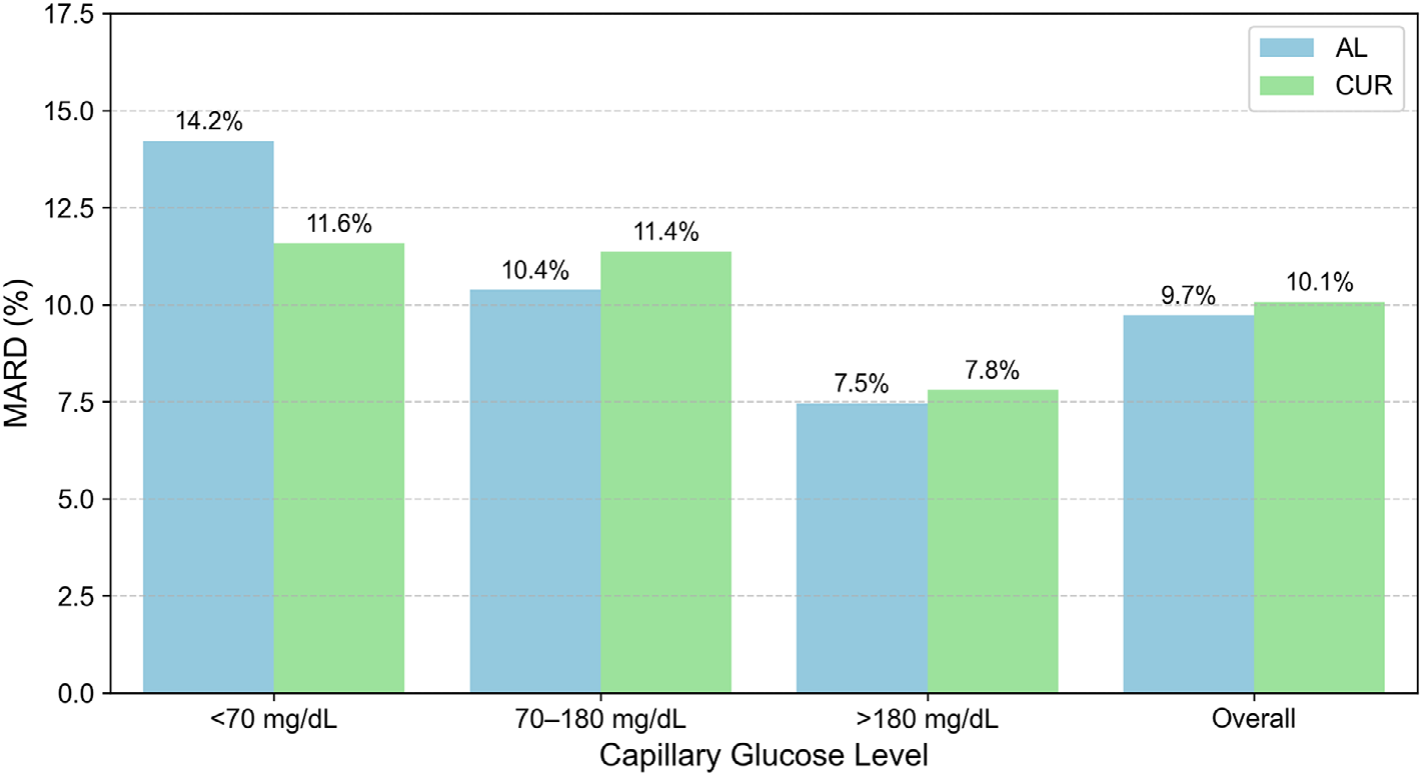
MARD by glycaemic range for AL and CUR glucose values, using capillary blood glucose as reference. Bars show MARD values across hypoglycaemic (<70 mg/dL), euglycaemic (70–180 mg/dL), and hyperglycaemic (>180 mg/dL) ranges, as well as overall.

To further assess the clinical relevance of differences between CUR and AL values, the MARD was calculated against capillary blood glucose measurements, stratified by glycaemic range (Figure 3). MARD was highest in the hypoglycaemic range (<70 mg/dL) and lowest in the hyperglycaemic range (>180 mg/dL) for both AL and CUR values. Compared to AL values, CUR values showed a lower MARD in the hypoglycaemic range but a higher MARD in the euglycaemic and overall ranges. A similar trend was observed using venous reference values, shown in Appendix S1.

**FIGURE 4:**
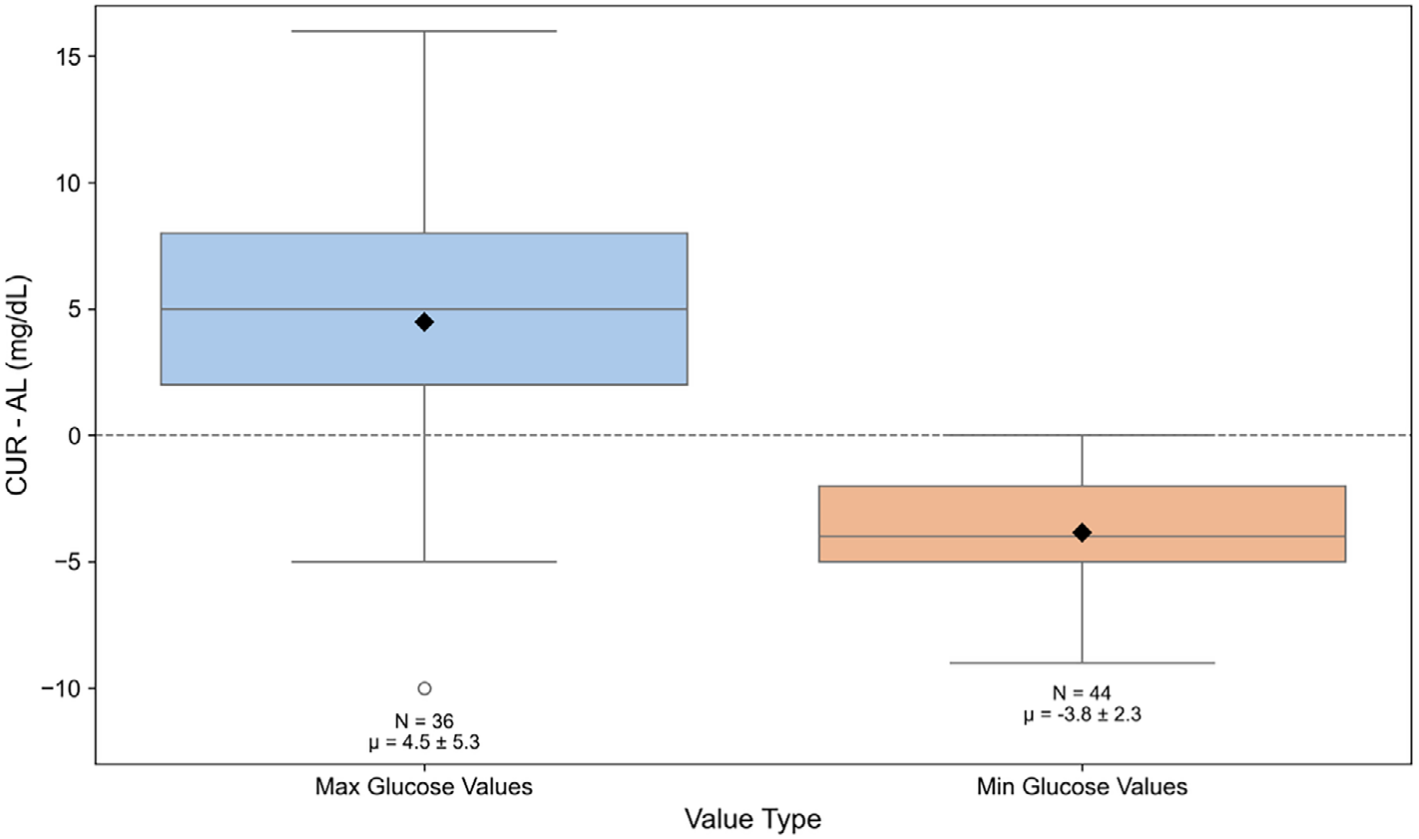
Differences between CUR and AL glucose values (CUR–AL) at matched maximum and minimum values during in‐clinic sessions. Only pairs within ±5 min were included.

To further characterise differences between CUR and AL data streams, we analysed peak glucose values during in‐clinic sessions. As shown in Figure 4, maximum glucose values tended to be slightly higher in CUR than in AL (mean difference: +4.5 ± 5.3 mg/dL), while minimum glucose values were slightly lower in CUR than in AL (mean difference: −3.8 ± 2.3 mg/dL). These modest differences suggest that AL values may not capture all extreme CUR values.

### 3.3 Display errors

**FIGURE 5:**
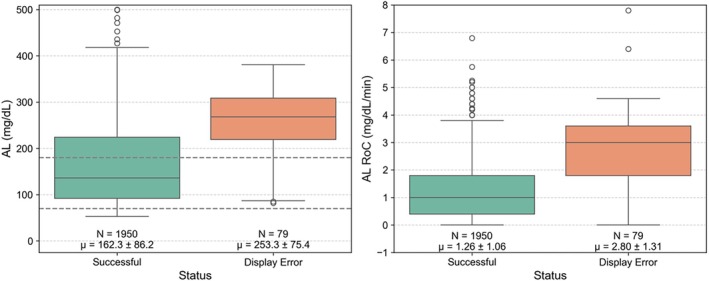
Comparison of AL and AL RoC glucose values at CUR retrieval attempts with and without display errors. Boxes represent the interquartile range (25th to 75th percentile) with medians as horizontal lines. Whiskers extend to 1.5 times the interquartile range; outliers are shown as individual points. Diamonds denote mean values. The dashed lines indicate glucose thresholds for hypo‐ and hyperglycaemia, mean, standard deviation, and number of points are annotated for each category.

To assess study conditions associated with display errors, glucose levels and RoC were compared between CUR retrieval attempts labelled as ‘Successful’ and ‘Display Error’. Figure 5 displays the distribution of AL glucose values (left) and RoC (right) at the time of each CUR retrieval attempt, grouped by retrieval outcome. Glucose levels were markedly higher during CUR retrieval attempts labelled as display errors, with few observations in the hypoglycaemic range. Likewise, absolute RoC values were elevated during these attempts, reflecting more rapid glucose fluctuations. Mixed‐effects models confirmed that display errors were associated with significantly higher glucose levels (mean difference: +91.9 mg/dL, *p* < 0.001) and greater rates of glucose change (mean difference: +1.52 mg/dL/min, *p* < 0.001), after accounting for repeated measures within participants.

## 4 DISCUSSION

This study examined the relationship between CUR and AL values from the FSL3 system, focusing on differences across glycaemic ranges and the circumstances under which display errors occur. While absolute differences between the two data streams were generally small, they showed systematic patterns, particularly in hypoglycaemia and during rapid glucose fluctuations.

In clinical practice, both individuals with diabetes and healthcare providers often assume that CUR and AL values can be used interchangeably. However, small differences between these two modalities are expected and should be interpreted considering the glycaemic context. In hypoglycaemia, CUR was slightly lower than AL with lower MARD (closer to the reference); accordingly, reliance on AL alone may under‐represent hypoglycaemia perceived by users. In contrast, the slightly greater variability observed in euglycaemia and hyperglycaemia is unlikely to affect most therapeutic decisions but may contribute to uncertainty when precise dosing is required. Importantly, clinicians typically review AL values in retrospective reports (e.g., LibreView), whereas patients act on CUR values in real time. This mismatch may lead to confusion during consultations if the causes for patient‐reported actions, such as treatment of hypoglycaemia after the display of a CUR value <70 mg/dL, are not reflected in the AL data. Notably, discrepancies exceeding ±10 mg/dL are expected to occur about once per day under typical frequencies of CUR retrieval. Recognising this pattern may help to avoid overreactions to isolated outlier readings. If MARD is taken as a proxy for accuracy, AL values appear more reliable overall and in hyperglycaemia, while CUR values are slightly more accurate in hypoglycaemia. Although average biases were small, their direction varies by range and they coincide with clinically critical contexts (hypoglycaemia, rapid excursions), which can influence patient actions and clinic interpretation.

Prior studies have also reported discrepancies between scanned and stored CGM values in earlier‐generation systems. For example, Pleus et al.^9^ analysed differences in the FreeStyle Libre 1 system and found that scanned values tended to be higher than stored values, particularly during hypoglycaemia. While the direction of bias differs from the present findings, both studies underscore the presence of systematic differences between CGM data streams. These discrepancies appear to be influenced by glycaemic context and may reflect changes in sensor design, signal processing, or data retrieval protocols across device generations.

These findings also raise important questions about how CGM data streams are processed and used in automated insulin delivery (AID) systems. Manufacturers have not disclosed whether AID systems rely on the AL or CUR values, nor the extent of any post‐processing applied (e.g., moving averaging). The smoother appearance of AL traces suggests that some signal filtering may be in place, potentially influencing control decisions. A systematic mismatch between CUR and AL values could lead to diverging behaviours between users and automated systems if the latter relies on AL values. In hypoglycaemia, lower CUR values (with lower MARD) may prompt appropriate treatment that is not evident in AL‐based reports, creating discrepancies during follow‐up. Conversely, during hyperglycaemia, a positively biased CUR value may prompt the user to deliver a correction dose, while the AID system might interpret the AL data differently and withhold action. Such inconsistencies may not only result in inappropriate therapy decisions but could also undermine trust in automated systems.

Display errors, while infrequent, occurred predominantly during periods of high glucose concentrations and rapid glucose excursions, conditions under which users are more likely to initiate glucose measurements. These findings highlight the need for device robustness during clinically critical moments, as missed or failed measurements under such conditions could delay the detection of hypo‐ or hyperglycaemia, potentially undermining user confidence in the system. During periods of rapid glucose change, confirmatory point‐of‐care capillary blood glucose testing may be beneficial, given interstitial–capillary lag and the higher likelihood of display errors under these conditions. In addition, display errors increase the need for confirmatory BG measurements. A key limitation of this study is that only scheduled CUR retrievals performed by study personnel were analysed. In routine use, individuals are more likely to initiate CUR retrievals during hypoglycaemia or rapid glucose fluctuations, conditions under which discrepancies and display failures may be more frequent, suggesting that the clinical impact could be underestimated in this controlled setting. Another limitation is the relatively small cohort size (*n* = 24), although each participant completed three in‐clinic sessions, resulting in 1950 CUR retrievals. Confirmation in larger cohorts would be valuable.

Overall, the observed differences between current displayed and auto‐logged values were, on average, minor but not negligible, and potentially important in some situations. Understanding these nuances may support better alignment between user decisions, provider interpretation, and automated systems and ultimately improve confidence in CGM use for daily diabetes care. The rationale for maintaining two distinct CGM data streams remains unclear and may reflect historical design choices rather than current clinical needs.

## AUTHOR CONTRIBUTIONS

LW and CM performed data analysis and statistical modelling. ME, DW, SP, and GF conceived and designed the study. ME, DW, and GF collected and curated the clinical CGM data. LW drafted the initial manuscript. CM, JG, and ME contributed to interpreting results. All authors provided critical revisions and approved the final version.

## FUNDING INFORMATION

Financial support to partially cover the costs of this study was provided by BIONIME Corporation, Diabetes Center Berne, University of Bern, i‐SENS, Inc., and Roche Diabetes Care GmbH. In addition, Ascensia Diabetes Care Holdings AG provided blood glucose monitoring systems and associated consumables free of charge. None of the commercial entities had any influence on the study design, data analysis, presentation, or publication of results. The remaining costs were carried by the Institute for Diabetes Technology. No funding was provided by the manufacturer of the examined CGM systems.

## CONFLICT OF INTEREST STATEMENT

The authors declared the following potential conflicts of interest with respect to the research, authorship, and/or publication of this article: GF is the general manager and medical director of the Institute for Diabetes Technology (Institut für Diabetes‐Technologie Forschungs‐ und Entwicklungsgesellschaft mbH an der Universität Ulm, Ulm, Germany), which carries out clinical studies, for example, with medical devices for diabetes therapy on its own initiative and on behalf of various companies. GF/IfDT have received research support, speakers' honoraria, or consulting fees in the last three years from Abbott, Ascensia, Bionime, Boydsense, Dexcom, Insulet, Lilly, Novo Nordisk, Perfood, Pharmasens, Roche, Sinocare, Terumo, Vertex, and Ypsomed. ME, SP, and DW are employees of IfDT. The other authors have no conflicts of interest to declare.

## PEER REVIEW

The peer review history for this article is available at https://www.webofscience.com/api/gateway/wos/peer‐review/10.1111/dom.70140.

## Supporting information


**Appendix S1:** Supporting information.

## Data Availability

The data that support the findings of this study are available on request from the corresponding author. The data are not publicly available due to privacy or ethical restrictions.
