# Supplementary material for: Discrepancies between current displayed and auto‐logged glucose values in FreeStyle Libre 3: Implications for clinical interpretation
Source: Diabetes Obes Metab. 2025 Sep 18;27(12):7367–73. doi: 10.1111/dom.70140 (PMC12587223; doi:10.1111/dom.70140)
Supplement: Supplementary file 1 — Appendix S1: Supporting information. [file DOM-27-7367-s001.docx]

**Supplementary Appendix**

**Discrepancies Between Current Displayed and Auto-Logged Glucose Values in FreeStyle Libre 3: Implications for Clinical Interpretation**

Lilian Witthauer et al.

**S1 Differences between CUR and AL as a Function of RoC**

**
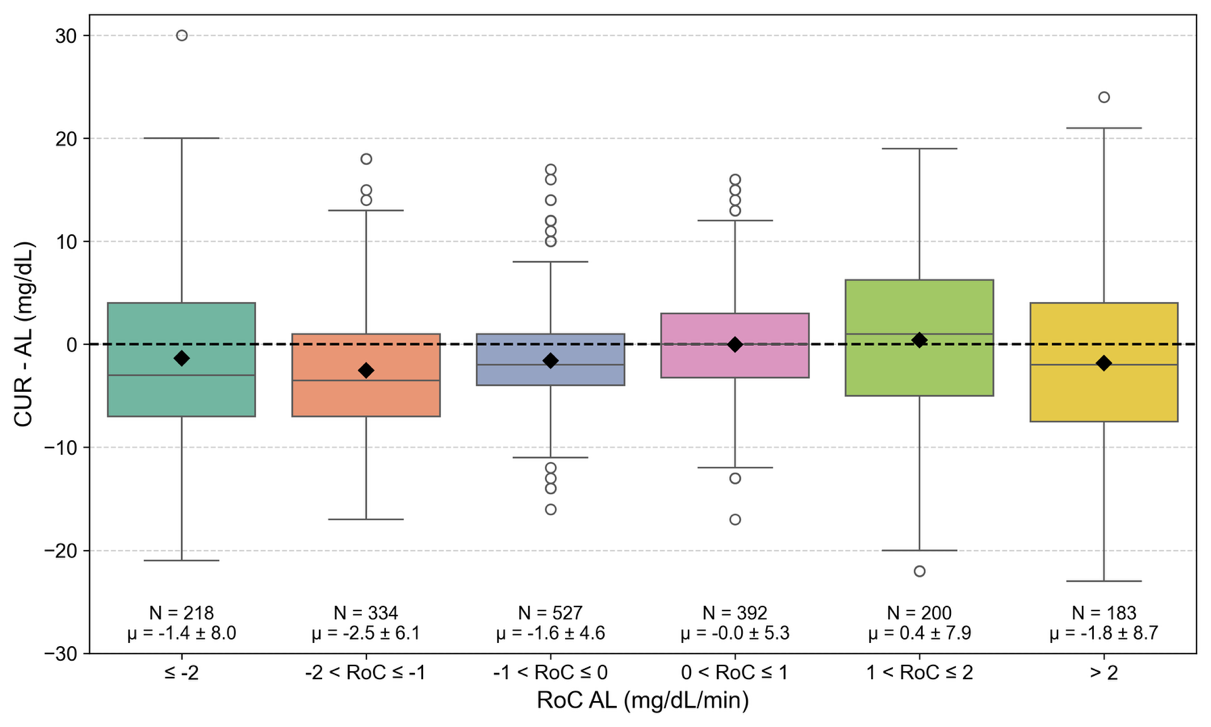
**

**S2 Shapiro-Wilk Test for Normality**

| **Glycemic Range** | **N** | **W Statistic** | **p-value** |
| --- | --- | --- | --- |
| <70 mg/dL | 181 | 0.973 | 0.002 |
| 70–180 mg/dL | 1031 | 0.990 | < 0.0001 |
| >180 mg/dL | 642 | 0.993 | 0.006 |
| Overall | 1854 | 0.988 | < 0.0001 |

**S3 Wilcoxon signed-rank test (CUR vs. AL):**

| **Glycemic Range** | **N** | **W Statistic** | **p-value** |
| --- | --- | --- | --- |
| <70 mg/dL | 181 | 452 | < 0.0001 |
| 70–180 mg/dL | 1031 | 144,920 | < 0.0001 |
| >180 mg/dL | 642 | 85,782 | 0.1935 |
| Overall | 1854 | 559,528 | < 0.0001 |

**S4 MARD Values for Current Displayed Values and Auto-logged Values (Venous Blood Glucose)**

**
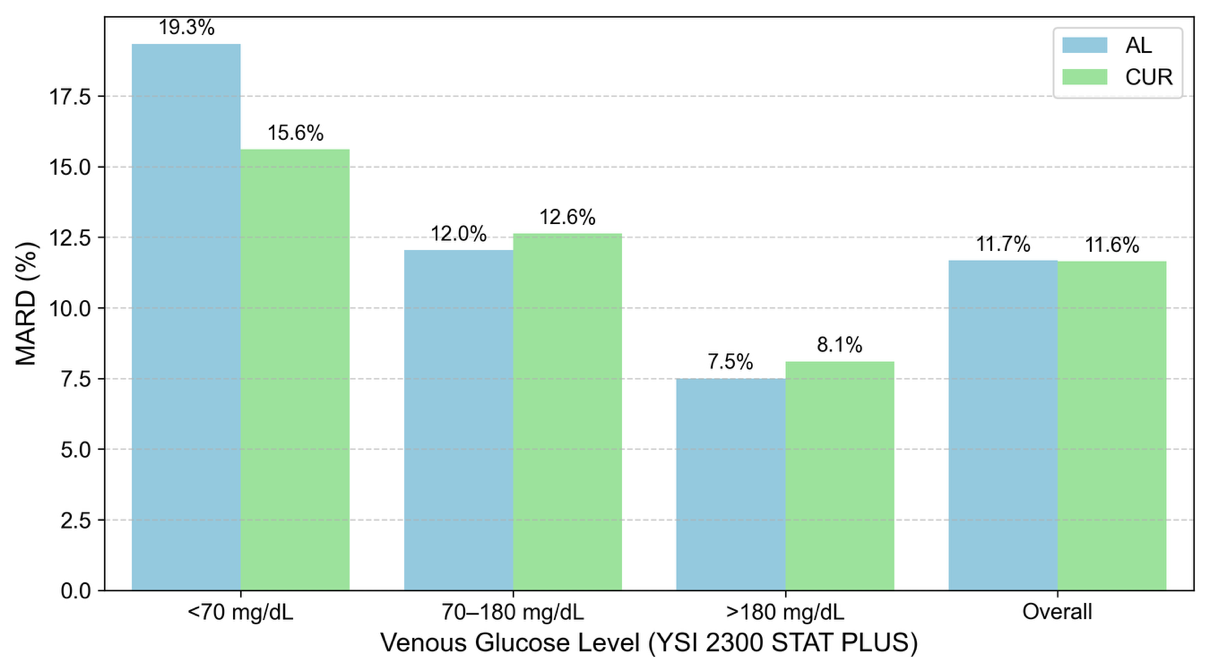
**

**
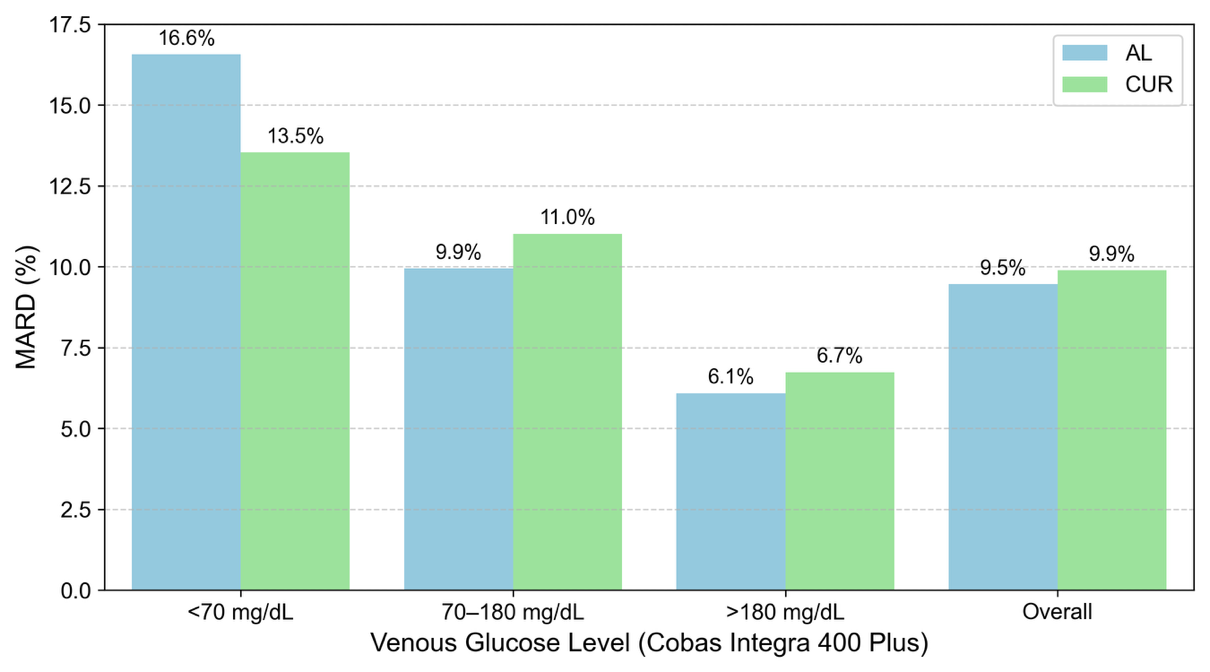
**

**S5 Mixed-Effects Model Results Comparing ‘Successful’ and ‘Display Error’ Scans**Mixed-effects models with participant ID as random effect. The outcome variable was AL glucose (mg/dL) or absolute rate of change (mg/dL/min). “Successful” indicates CUR values were displayed; “Display Error” served as the reference category. Negative coefficients therefore indicate lower values during Successful scans compared with Display Errors.

| **Outcome Variable** | **Coefficient for CUR ‘Successful’** | **95% CI (Lower–Upper)** | **p-value** |
| --- | --- | --- | --- |
| Glucose (AL) | −91.9 mg/dL | −111.0 to −72.8 mg/dL | <0.001 |
| Rate of Change (RoC) | −1.52 mg/dL/min | −1.76 to −1.28 mg/dL/min | <0.001 |
